# 

**Published:** 1895-08

**Authors:** 


					﻿THE
Southern Medical Record.
A Monthly Journal of Medicine and Surgery.
Vol. XXV.	ATLANTA, GA., AUGUST, 1895.	No. 8?
BERNARD WOLFF, M. D., Editor and Proprietor.
Associate Editors :
A. W. GRIGGS, M. D.
j. mcfadden gaston, m. d.
WILLIS F. WESTMORELAND, M. D.
LOUIS H. JONES, M. D.
Entered at the Post-office at Atlanta, Ga., as Second-Class Matter.
The Eoote &' Davies Co., Atlanta, Ga.
				

